# Acridine orange fluorescent microscopy is more sensitive than India ink light microscopy in the rapid detection of cryptococcosis among CrAg positive HIV patients

**DOI:** 10.1371/journal.pone.0182108

**Published:** 2017-07-27

**Authors:** Richard Kwizera, Andrew Akampurira, Darlisha Williams, David R. Boulware, David B. Meya, on behalf of the ASTRO-CM Study Team

**Affiliations:** 1 Infectious Diseases Institute, College of Health Sciences, Makerere University, Kampala, Uganda; 2 Division of Infectious Diseases and International Medicine, Department of Medicine, University of Minnesota, Minneapolis, Minnesota, United States of America; 3 Department of Medicine, School of Medicine, College of Health Sciences, Makerere University, Kampala, Uganda; OSWALDO CRUZ FOUNDATION, BRAZIL

## Abstract

**Background:**

India ink microscopy on cerebrospinal fluid is still utilized in resource limited settings for the diagnosis of cryptococcal meningitis despite its poor sensitivity. We hypothesized that staining fungal nucleic acids with fluorescent dyes instead of the capsule with India ink might improve sensitivity for the diagnosis of cryptococcal meningitis.

**Methods:**

We enrolled 96 HIV-infected participants with cryptococcal meningitis who provided 194 CSF specimens at serial time points in Kampala, Uganda. Cryptococcosis was diagnosed by cerebrospinal fluid (CSF) cryptococcal antigen (CrAg) test and only positive samples were included. We stained CSF with India ink and acridine orange. We cultured the same samples on standard fungal media. We compared acridine orange to CrAg, India ink and CSF culture.

**Results:**

Acridine orange was more sensitive (96%) than India ink (79%) with reference to CSF CrAg. Acridine orange and India ink had a statistically significant difference (P<0.001) with a 25% correlation for detection of *Cryptococcus* yeasts. India ink had more negative results (22%) than acridine orange (4%). The sensitivity for India ink increased (86%) while that of acridine orange did not change (97%) when compared to CSF culture. However, both India ink and acridine orange had poor predictive values with reference to culture.

**Conclusion:**

Acridine orange is a better alternative to India ink in the rapid detection of cryptococcosis among CrAg positive HIV patients.

## Introduction

Cryptococcal meningitis (CM) is the most common cause of adult meningitis in Africa accounting for 15%–25% of AIDS-related deaths; with majority of the cases registered in sub-Saharan Africa [1–4]. Culture of cerebrospinal fluid (CSF) is the gold standard for diagnosis of CM. However, detection of the cryptococcal antigen (CrAg) in CSF, serum and whole blood has recently gained more popularity with better sensitivity [5, 6].

India ink has over time been used in the rapid diagnosis of cryptococcal meningitis using cerebrospinal fluid (CSF) among AIDS patients [7]. It is still being used in some resource limited settings of sub-Saharan Africa where the burden due to cryptococcal meningitis is highest. However, many recent studies done in sub-Saharan Africa in clinical settings demonstrated low and poor sensitivity of India ink in the diagnosis of cryptococcal meningitis [8, 9]. The new point of care CrAg lateral flow assay is in fact more sensitive and user friendly in different sample types [4, 6, 8, 10]. The poor sensitivity of India ink may lead to misdiagnosis hence increasing the burden and mortality due to cryptococcal meningitis in resource limited settings where it is still utilized.

Acridine orange is a fluorescence dye used to stain acidic vacuoles, DNA, and RNA in viable cells. It has been used to stain samples for viability staining, epifluorescence microscopy and is particularly useful in the rapid screening of sterile biological specimens from clinical and non-clinical materials [11]. It emits yellow fluorescence when it binds RNA and green fluorescence when it binds DNA. Earlier work done by Cohen et al, 1984 showed no apparent advantage of acridine orange over india ink for the rapid identification of *Cryptococcus neoformans* using three laboratory strains [12].

In this study, we used acridine orange to detect cryptococcal yeasts in fresh whole CSF in comparison to India ink stain at serial time points during antifungal treatment in a clinical setting. We hypothesized that with the availability of a florescent microscope in a clinical laboratory, acridine orange may be a more sensitive alternative to India ink light microscopy in the detection of *Cryptococcus* yeasts in CSF.

## Materials and methods

### Study population

The study population included patients diagnosed with cryptococcal meningitis at Mulago National Referral Hospital in Kampala, Uganda during the Adjunctive Sertraline for the Treatment of HIV-Associated Cryptococcal Meningitis (ASTRO) Clinical Trial, (ClincalTrials.gov: NCT01802385) [13]. Participants included in the study were HIV-infected, ≥18 years, with a positive CSF CrAg. CSF was collected at days 0,3,7,10, and 14 of cryptococcal meningitis treatment and as clinically indicated by therapeutic lumbar puncture as part of standard care.

### Ethical statement

All research participants or their surrogate provided written informed consent. Ethical approval was obtained from the relevant ethical review boards at Mulago National Referral Hospital in Uganda and at the University of Minnesota.

### Study procedures

For this study, Cryptococcal meningitis was confirmed using CSF cryptococcal antigen (CrAg) lateral flow assay (Immy, Norman, Oklahoma, USA). Following informed consent, only patients with a positive CSF CrAg were included for this diagnostic evaluation. Symptomatic HIV patients being managed for cryptococcal meningitis had lumbar punctures performed at serial time points. Acridine orange florescent staining was performed on fresh whole CSF samples (n = 194). India ink light microscopy was also performed on the same samples. For both tests, the level of positivity was graded on a scale of 0–3 where; zero = negative, +1 = 1–9 cells in 100 fields, +2 = 10–99 cells in 100 fields and +3 ≥100 cells in one field. Acridine orange florescent microscopy was compared to India ink light microscopy. We also compared both procedures to the gold standard (CSF culture). Fungal cultures on whole CSF were performed and incubated at 30°C for up to 10 days on Sabouraud dextrose agar.

### Acridine orange staining

Whole CSF was span at 10000g for 5 minutes. A 1:2 dilution of the CSF sediment was made in acridine orange dye in a cryovial (one drop [40μl] of CSF sediment added to one drop [40μl] of acridine orange). The concentration of acridine orange solution used was “0.1g acridine orange in 1000ml of 0.5M acetate buffer”. The mixture was left to stand for 5 minutes at room temperature. One drop of the mixture was then loaded onto a glass slide and a cover slip added. The slide was examined on a fluorescent microscope using a blue filter and 40x objectives.

### India ink staining

For this procedure, whole CSF was span for 5 minutes at 10000g. One drop (40μl) of India ink was put on a clean slide and one drop (40μl) of CSF sediment was added and the slide was examined using a light microscope through 40x objective.

### Statistical analysis

Statistical analysis was aimed at comparing the sensitivity, level of agreement and deviation in the levels of positive results obtained using acridine orange compared with India ink in the detection of cryptococcal cells at 95% confidence interval (CI). Data were analyzed using STATA version 13 (STATA, College Station, Texas).

## Results and discussion

### Characteristics of the study population

We enrolled 96 study participants with cryptococcal meningitis who provided 194 CSF specimens at serial time points in Kampala, Uganda from March 2015 to December 2015. 62% of participants were males with an overall median age of 35 years (interquartile range [IQR]: 28, 40). All participants were HIV-positive adult Ugandans with a median CD4 T cell count of 14 cells/μL (IQR: 5, 50) at diagnosis. Only 51% (49/96) of the participants were receiving antiretroviral therapy at diagnosis. Among patients reporting a headache (n = 92), the median duration of headache was 14 days (IQR: 7, 30). The CSF opening pressures at baseline (n = 95) had a median of 322 mmH_2_O (IQR: 210, 440) while the median CSF white blood cell count was 4 cells/mL (IQR: 4, 45) (**Table 1**).

**Table 1:** Baseline characteristics of the study population.

BASELINE CHARACTERISTICS	N	STATISTIC
Males*, n (%)	90	56 (62)
Age* (years), median(IQR)	90	35 (30, 40)
On ART, n (%)	96	49 (51)
CD4 T-cell* (cells/mL), median(IQR)	92	14 (5, 51)
Headache, n (%)	96	92 (95.8)
Headache duration* (days), median (IQR)	92	14 (7, 30)
CSF Opening pressures* (mmH_2_O), median(IQR)	95	322 (210, 440)
CSF WBC's/mL*, median (IQR)	89	4 (4, 45)

Data presented are percentages (%), medians and interquartile ranges (IQR). ART = antiretroviral therapy, CSF = cerebrospinal fluid, WBC = white blood cells, N = number of participants with data for each parameter.

*Some parameters have N<96 due to missing data.

### Performance of India ink and acridine orange against culture and CrAg

Of the 194 CrAg LFA positive CSF samples tested from different time points during antifungal therapy, 152 were positive for India ink, 186 were positive for acridine orange, and 151 were positive for quantitative cultures. 127 samples were positive for all these three tests. The overall (n = 194) median for quantitative cultures was 820 CFU/ml (IQR = 10 to 73000). Acridine orange had a sensitivity of 96% while India ink had a sensitivity of 78% with reference to CSF CrAg (n = 194).

For both India ink and acridine orange tests, the level of positivity was graded on a scale of zero (0) to +3 where; zero = negative, +1 = 1–9 cells in 100 fields, +2 = 10–99 cells in 100 fields and +3 is ≥100 cells in one field. Using India ink (n = 194), 33% of the samples had a low positivity grade of +1, followed by a +3 (25%), then zero (negative—22%) and the least with a +2 (21%). However, using acridine orange (n = 194), 47% of the samples had a positivity grade of +1, followed by a +2 (29%), then +3 (20%) and the least with zero (negative- 4%) (**Table 2**).

**Table 2:** Performance of India ink and Acridine orange against culture.

		n (number that were culture positive)				
	Grade of positivity	Negative	+1	+2	+3	Total
**TEST**	**India ink**	42 (22)	63 (46)	41 (36)	48 (47)	194 (151)
	**Acridine orange**	8 (5)	91 (72)	56 (43)	39 (31)	194 (151)

Data presented are number of samples (n) for the different grades of positivity and the number of samples that were positive for culture (gold standard) in each category. All samples were CrAg positive.

Using the fishers exact test, there was a statistically significant difference (p<0.001) in the grades of positivity between acridine orange and India ink at a 95% CI. However, there was a 25% correlation between the two tests. The same statistical analysis also revealed that acridine orange was more sensitive (96%) than India ink (79%) with reference to CSF CrAg. This confirmed the analysis done in the previous paragraphs. In addition, since all samples were from known positive cases (using CSF CrAg), this result was also supported by the fact that India ink had more negative results ([42] 22%) than acridine orange ([8] 4%).

**Fig 1:**
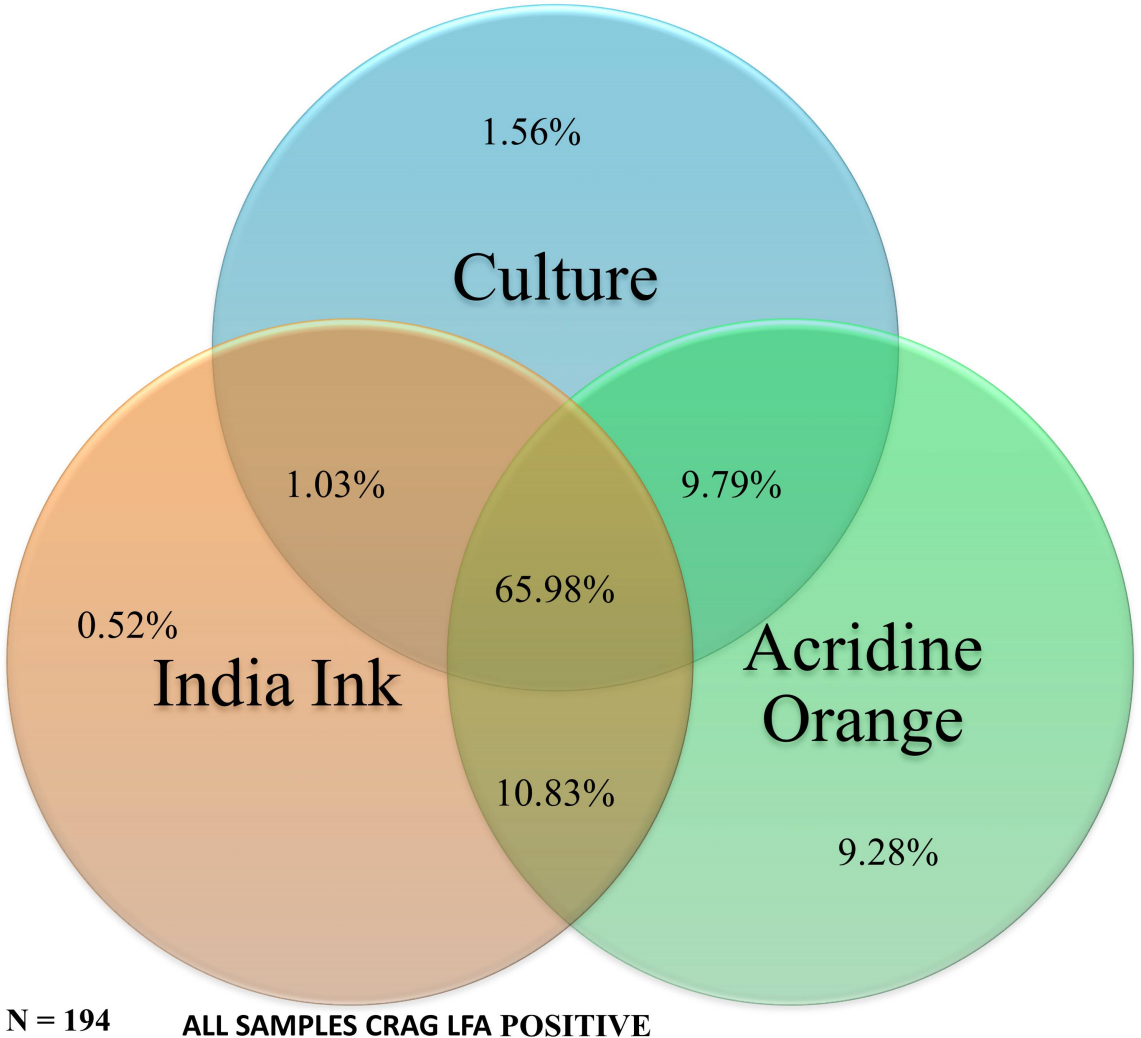
Venn diagram of the distribution of Cryptococcal meningitis CSF diagnostics. Distribution of 194 CSF samples from 96 cryptococcal meningitis cases. All samples were positive for CSF cryptococcal antigen lateral flow assay. Only 66% of the samples were positive for India ink, acridine orange and culture.

When compared to quantitative CSF culture, the sensitivity for India ink increased to 86% (130/152) while that of Acridine orange did not change (97%). However, both India ink (positive predictive value [PPV] = 86%, negative predictive value [NPV] = 48%) and acridine orange (PPV = 79%, NPV = 38%) had poor predictive values with reference to culture. 52% (22/42) of the India ink negative samples were positive with culture while 63% (5/8) of the acridine orange negative samples were positive with culture. When comparing both India ink and acridine orange to culture, false positive results were registered more among samples with a (+1) grade of positivity (**Table 2**). False negatives were registered more among patients who had received more than seven doses of Amphotericin B. However, with reference to CSF CrAg, CSF culture had a lower sensitivity (78.4%) and only 66% of the samples (n = 194) were positive for all the three tests (**Fig 1**). Even though culture of CSF is still considered the gold standard for diagnosis of cryptococcal meningitis, recent evidence indicates that detection of the cryptococcal antigen (CrAg) in CSF has a better sensitivity [5, 6].

**Fig 2:**
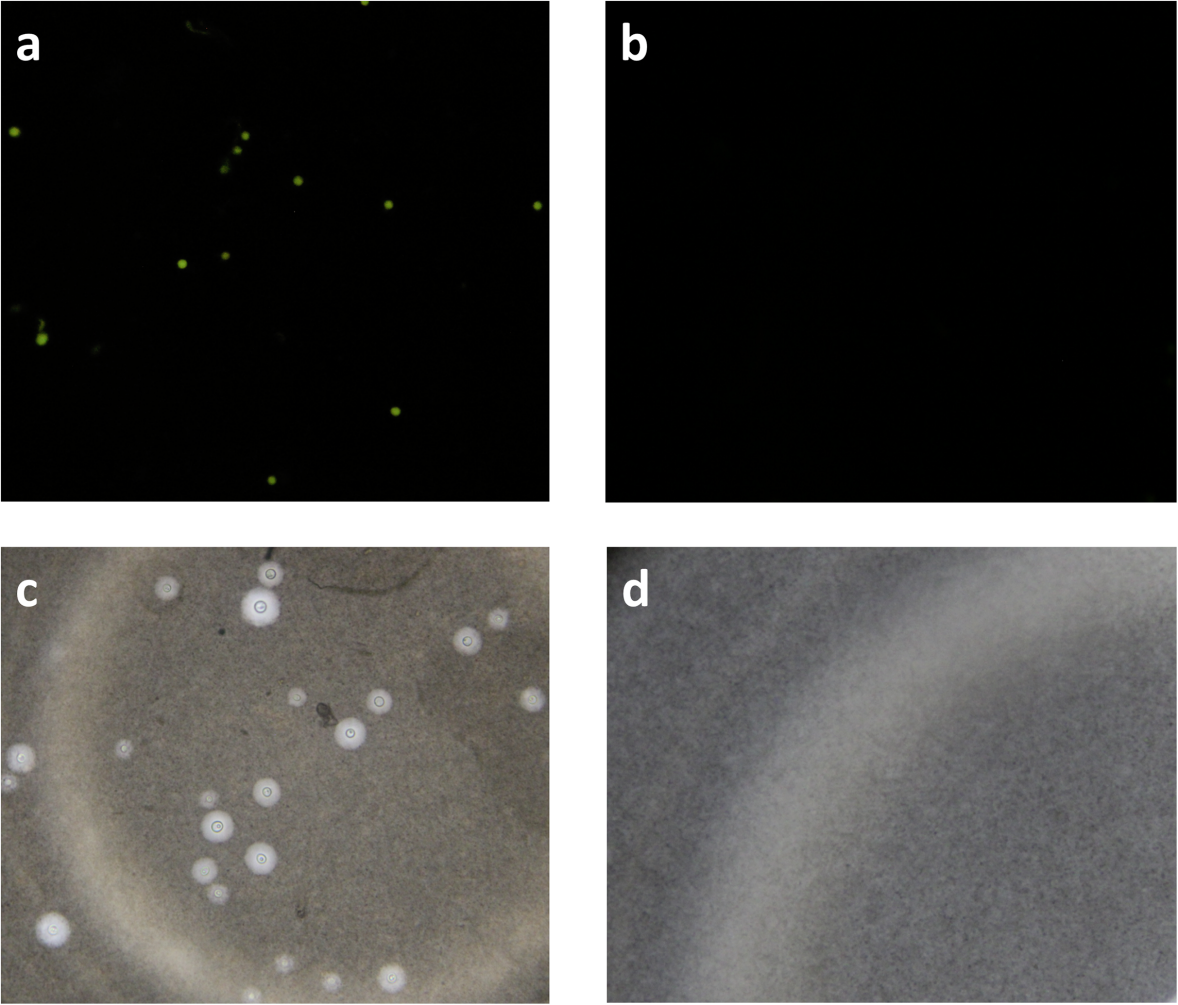
*Cryptoccocus* yeasts stained with acridine orange and India ink. (a) Image of a positive acridine orange slide showing *Cryptococcus* yeasts fluorescing green against a dark background, using a blue filter on a fluorescent microscope. (b) Image of a negative acridine orange slide showing no *Cryptococcus* yeasts. (c) Image of a positive india ink slide showing a capsule of *Cryptococcus* yeasts on a bright field light microscope. (d) Image of a negative india ink slide showing no *Cryptococcus* yeasts. All images were taken at 40x magnification.

These results suggest that fluorescent microscopy maybe more sensitive and reliable in mass screening programs for cryptococcal disease among HIV patients, especially in resource limited setting where the disease remains a burden to the health systems. Our prior utility of auramine-O staining for the detection of *Mycobacterium tuberculosis* among HIV patients indicated that fluorescent microscopy is more sensitive than light microscopy (*unpublished*). Therefore, at the beginning of the study we stained cryptococcal isolates with auramine-O. The cryptococcal yeasts were clearly visible fluorescing against a pale background. This differential fluorescence of cryptococcal yeasts picked our interest to evaluate acridine orange as a potential replacement for India ink. We thought that its ability to freely enter dead or live cells and bind to nucleic acids would be very vital and increase the sensitivity of determining sterility of cerebrospinal fluid rapidly other than using india ink to stain the capsule (**Fig 2**); which sometimes is absent in some strains among our HIV patients being managed for cryptococcal meningitis.

A similar study by Cohen investigated the sensitivity of India ink, acridine orange and latex agglutination for the rapid identification of *Cryptococcus neoformans*, by determining the limit of detection of three strains. Results indicated that conventional methods are of comparable sensitivity. However, there was no apparent advantage to the acridine orange method over the other tests [12]. Besides, acridine orange is a non-specific stain. Other fungal specific fluorescent dyes have been used before to stain fungi in human and animal tissue with good performance [14]. Most of them have been experimented on industrial yeasts to check for their viability [15, 16] and those used in clinical studies have been mainly used on respiratory samples and biopsies [17]. However, these are not readily available in our resource limited setting. They may actually provide a better alternative when introduced. Given the usual delays in diagnosis by culture [18] and low sensitivity by india ink [18, 19], acridine orange may provide a rapid alternative towards establishing a diagnosis to enable better treatment.

## Conclusion

The major study limitation was that we did not include samples that were negative for CSF CrAg. However, based on our findings, we conclude that with the availability of a fluorescent microscope, acridine orange is a better alternative to India ink in the rapid detection of cryptococcosis among CrAg positive HIV patients especially in sub-Saharan Africa where the disease remains a burden to health systems.

## Supporting information

S1 Table Full data set. Spreadsheet contains all raw data for the study population.
(XLSX)
